# Supplementary material for: Profiling of Circulating microRNAs in Prostate Cancer Reveals Diagnostic Biomarker Potential
Source: Diagnostics (Basel). 2020 Mar 28;10(4):188. doi: 10.3390/diagnostics10040188 (PMC7235761; doi:10.3390/diagnostics10040188)
Supplement: Supplementary file 1 [file diagnostics-10-00188-s001.pdf]

$$\log\left(\frac{p(malignancy)}{1-p(malignancy)}\right) = 5.407 + 0.474 \times bCaP + 0.045 \times PSA$$

$$\log\left(\frac{p(malignancy)}{1-p(malignancy)}\right) = 7.103 + 0.629 \times bCaP + 0.026 \times PSA + 1.984 \times \mathrm{DRE} - 0.006 \times age$$

Supplementary Table 1. Coefficients for the logistic regression model for combining *bCaP* + PSA, or *bCaP* + PSA + DRE + Age. PSA: prostate specific antigen; DRE: digital rectal examination.

**Upregulated in APC**	**Fold change**	***p* value**	**BH corrected *p* value**	**Downregulated in APC**	**Fold change**	***p* value**	**BH corrected *p* value**
**hsa-miR-26a-5p**	1.89	5.46E-12	2.51E-10	**hsa-miR-146a-5p**	-1.73	1.75E-12	1.61E-10
**hsa-miR-30c-5p**	1.75	2.47E-10	7.58E-09	**hsa-miR-24-3p**	-1.32	5.66E-07	6.51E-06
**hsa-miR-142-3p**	1.89	2.78E-09	6.39E-08	**hsa-miR-130a-3p**	-1.48	2.62E-06	2.19E-05
**hsa-miR-30b-5p**	1.64	4.04E-09	7.44E-08	**hsa-miR-221-3p**	-1.30	8.62E-06	4.95E-05
**hsa-miR-375**	3.70	2.49E-07	3.44E-06	**hsa-miR-199a-3p**	-1.42	8.91E-05	3.04E-04
**hsa-miR-145-5p**	1.53	2.61E-07	3.44E-06	**hsa-miR-197-3p**	-1.26	1.75E-04	5.55E-04
**hsa-miR-25-3p**	1.46	7.53E-07	7.69E-06	**hsa-miR-130b-3p**	-1.33	2.04E-04	6.25E-04
**hsa-let-7d-5p**	1.57	2.36E-06	2.17E-05	**hsa-miR-23a-3p**	-1.17	3.78E-04	1.05E-03
**hsa-miR-215-5p**	1.75	3.33E-06	2.36E-05	**hsa-miR-410-3p**	-1.59	6.93E-04	1.66E-03
**hsa-miR-106b-5p**	1.42	3.26E-06	2.36E-05	**hsa-miR-376c-3p**	-1.64	6.81E-04	1.66E-03
**hsa-miR-484**	1.37	6.03E-06	3.96E-05	**hsa-miR-27b-3p**	-1.13	1.29E-03	2.89E-03
**hsa-miR-331-3p**	1.51	7.54E-06	4.63E-05	**hsa-miR-154-5p**	-1.51	3.91E-03	7.89E-03
**hsa-miR-194-5p**	1.68	1.28E-05	6.95E-05	**hsa-miR-152-3p**	-1.21	6.50E-03	1.20E-02
**hsa-let-7c-5p**	1.54	1.57E-05	8.04E-05	**hsa-miR-22-5p**	-1.34	9.89E-03	1.69E-02
**hsa-miR-17-3p**	1.63	2.10E-05	1.01E-04	**hsa-miR-128-3p**	-1.27	1.06E-02	1.77E-02
**hsa-let-7f-5p**	1.53	2.28E-05	1.05E-04	**hsa-miR-27a-3p**	-1.12	1.66E-02	2.59E-02
**hsa-miR-451a**	1.84	2.76E-05	1.21E-04	**hsa-miR-376a-3p**	-1.41	2.53E-02	3.88E-02
**hsa-miR-140-3p**	1.39	3.33E-05	1.39E-04	**hsa-miR-584-5p**	-1.23	2.67E-02	4.03E-02
**hsa-let-7i-5p**	1.28	4.69E-05	1.88E-04				
**hsa-let-7a-5p**	1.38	6.15E-05	2.36E-04				
**hsa-miR-18b-5p**	1.27	6.87E-05	2.53E-04				
**hsa-miR-16-5p**	1.38	8.51E-05	3.01E-04				
**hsa-miR-181a-5p**	1.46	1.36E-04	4.47E-04				
**hsa-miR-210-3p**	1.48	3.06E-04	9.07E-04				
**hsa-miR-93-5p**	1.17	3.78E-04	1.05E-03				
**hsa-miR-363-3p**	1.42	4.02E-04	1.09E-03				
**hsa-miR-126-3p**	1.18	4.48E-04	1.18E-03				
**hsa-miR-18a-5p**	1.27	5.65E-04	1.44E-03				
**hsa-miR-486-5p**	1.49	7.03E-04	1.66E-03				
**hsa-let-7g-5p**	1.20	1.01E-03	2.33E-03				
**hsa-miR-16-2-3p**	1.34	1.45E-03	3.17E-03				
**hsa-miR-107**	1.53	2.16E-03	4.62E-03				
**hsa-miR-151a-5p**	1.20	2.96E-03	6.19E-03				
hsa-miR-29c-3p	1.29	3.94E-03	7.89E-03				
**hsa-miR-101-3p**	1.27	4.50E-03	8.80E-03				
**hsa-miR-19a-3p**	1.22	5.00E-03	9.58E-03				
**hsa-miR-660-5p**	1.29	5.45E-03	1.02E-02				
**hsa-miR-425-5p**	1.14	7.03E-03	1.27E-02				
hsa-miR-122-5p	1.60	8.64E-03	1.53E-02				

**hsa-miR-30e-5p**	1.14	9.61E-03	1.67E-02
hsa-miR-199a-5p	1.28	1.31E-02	2.14E-02
**hsa-miR-140-5p**	1.14	1.34E-02	2.16E-02
**hsa-miR-20a-5p**	1.14	1.38E-02	2.19E-02
**hsa-miR-19b-3p**	1.14	2.84E-02	4.22E-02

Supplementary Table 2. Dysregulated miRNAs between samples from BPH patients and samples from APC patients. Bold, underlined miRNAs overlap with miRNAs in Supplementary Table 2. BPH: benign prostatic hyperplasia; LPC: localized prostate cancer; APC: advanced prostate cancer.

Upregulated in APC	Fold change	*p* value	BH corrected *p* value
**hsa-miR-26a-5p**	2.55	5.10E-21	2.52E-19
**hsa-miR-25-3p**	1.96	5.48E-21	2.52E-19
**hsa-miR-30c-5p**	2.53	2.73E-20	6.27E-19
**hsa-miR-16-5p**	2.11	2.14E-20	6.27E-19
**hsa-miR-142-3p**	2.76	5.79E-18	1.06E-16
**hsa-miR-451a**	3.33	1.15E-17	1.76E-16
**hsa-miR-30b-5p**	2.11	3.33E-17	3.83E-16
**hsa-let-7d-5p**	2.23	1.44E-16	1.47E-15
**hsa-miR-486-5p**	2.34	2.61E-16	2.40E-15
**hsa-let-7i-5p**	1.60	8.28E-16	6.93E-15
**hsa-miR-363-3p**	2.10	9.85E-15	6.97E-14
**hsa-let-7f-5p**	2.12	3.76E-14	2.31E-13
**hsa-miR-210-3p**	1.92	1.82E-12	1.05E-11
**hsa-miR-140-3p**	1.71	2.66E-12	1.44E-11
hsa-miR-92a-3p	1.49	4.35E-12	2.23E-11
**hsa-miR-145-5p**	1.85	6.52E-12	3.16E-11
**hsa-miR-93-5p**	1.27	3.08E-11	1.35E-10
**hsa-miR-331-3p**	1.69	3.46E-11	1.45E-10
**hsa-let-7a-5p**	1.70	6.61E-11	2.65E-10
**hsa-miR-16-2-3p**	1.89	7.16E-11	2.74E-10
**hsa-let-7c-5p**	1.97	8.29E-11	3.05E-10
hsa-miR-15a-5p	1.66	2.45E-10	8.66E-10
**hsa-miR-425-5p**	1.32	4.42E-10	1.51E-09
**hsa-miR-107**	2.19	4.60E-10	1.51E-09
**hsa-miR-140-5p**	1.42	6.99E-10	2.22E-09
**hsa-miR-19b-3p**	1.33	8.69E-10	2.58E-09
**hsa-miR-20a-5p**	1.22	8.62E-10	2.58E-09
**hsa-miR-18a-5p**	1.40	4.61E-09	1.33E-08
**hsa-miR-181a-5p**	1.64	7.29E-08	1.97E-07
**hsa-miR-215-5p**	1.82	9.51E-08	2.50E-07
**hsa-miR-19a-3p**	1.30	1.57E-07	4.02E-07
**hsa-miR-18b-5p**	1.33	2.43E-07	6.03E-07
**hsa-miR-106b-5p**	1.31	7.59E-07	1.84E-06
**hsa-miR-151a-5p**	1.28	9.59E-07	2.26E-06
**hsa-miR-375**	3.27	1.59E-06	3.56E-06
**hsa-miR-126-3p**	1.24	1.73E-06	3.79E-06
**hsa-miR-484**	1.40	1.98E-06	4.15E-06
**hsa-miR-194-5p**	1.76	2.11E-06	4.31E-06
**hsa-miR-101-3p**	1.32	6.19E-06	1.14E-05
hsa-miR-185-5p	1.19	1.39E-05	2.37E-05
**hsa-miR-660-5p**	1.39	2.43E-05	4.06E-05
**hsa-let-7g-5p**	1.21	3.42E-05	5.62E-05
**hsa-miR-17-3p**	1.52	4.26E-05	6.87E-05
**hsa-miR-30e-5p**	1.16	1.24E-04	1.81E-04
hsa-miR-345-5p	1.21	1.03E-02	1.34E-02

Downregulated in APC	Fold change	*p* value	BH corrected *p* value
**hsa-miR-146a-5p**	-1.82	1.86E-17	2.45E-16
**hsa-miR-27a-3p**	-1.57	1.89E-15	1.45E-14
**hsa-miR-27b-3p**	-1.53	2.44E-14	1.60E-13
**hsa-miR-23a-3p**	-1.27	3.08E-11	1.35E-10
**hsa-miR-24-3p**	-1.30	6.62E-08	1.85E-07
hsa-miR-142-5p	-1.43	1.18E-06	2.70E-06
hsa-miR-328-3p	-1.45	1.94E-06	4.15E-06
**hsa-miR-199a-3p**	-1.47	2.57E-06	5.15E-06
**hsa-miR-197-3p**	-1.32	4.60E-06	8.93E-06
**hsa-miR-130a-3p**	-1.37	4.66E-06	8.93E-06
hsa-miR-29a-3p	-1.49	5.79E-06	1.09E-05
**hsa-miR-128-3p**	-1.48	9.24E-06	1.67E-05
**hsa-miR-221-3p**	-1.33	9.77E-06	1.73E-05
hsa-miR-150-5p	-1.60	1.36E-05	2.37E-05
hsa-miR-23b-3p	-1.20	4.91E-05	7.78E-05
**hsa-miR-376c-3p**	-1.71	5.21E-05	8.12E-05
**hsa-miR-376a-3p**	-1.69	5.96E-05	9.14E-05
**hsa-miR-410-3p**	-1.65	6.39E-05	9.64E-05
**hsa-miR-154-5p**	-1.69	6.84E-05	1.01E-04
**hsa-miR-22-5p**	-1.44	1.34E-04	1.92E-04
hsa-miR-369-3p	-1.71	1.66E-04	2.34E-04
**hsa-miR-152-3p**	-1.22	2.04E-04	2.85E-04
hsa-miR-382-5p	-1.53	1.88E-03	2.58E-03
hsa-miR-151a-3p	-1.15	6.08E-03	8.22E-03
hsa-miR-134-5p	-1.42	8.51E-03	1.14E-02
**hsa-miR-584-5p**	-1.25	9.24E-03	1.21E-02
hsa-miR-21-5p	-1.08	2.69E-02	3.44E-02
**hsa-miR-130b-3p**	-1.18	2.80E-02	3.53E-02

Supplementary Table 3. Dysregulated miRNAs between samples from LPC patients and samples from APC patients. Bold, underlined miRNAs overlap with miRNAs in Supplementary Table 1. BPH: benign prostatic hyperplasia; LPC: localized prostate cancer; APC: advanced prostate cancer.

Upregulated in PSA >= 10	Fold change	*p* value	BH corrected *p* value	Downregulated in PSA >= 10	Fold change	*p* value	BH corrected *p* value
hsa-miR-122-5p	1.39	0.00023	0.01054	hsa-miR-140-5p	-1.15	0.00020	0.01054
hsa-miR-29a-3p	1.15	0.00048	0.01112	hsa-miR-376c-3p	-1.22	0.00040	0.01112
hsa-miR-23a-3p	1.07	0.00092	0.01416	hsa-miR-93-5p	-1.07	0.00092	0.01416
hsa-miR-375	1.49	0.00159	0.01849	hsa-miR-376a-3p	-1.18	0.00161	0.01849
				hsa-miR-20a-5p	-1.06	0.00281	0.02876

Supplementary Table 4. Dysregulated miRNAs between samples from LPC patients with low PSA (≤10 ng/ml) versus high PSA (>10 ng/ml). LPC: localized prostate cancer; PSA: prostate specific antigen.

Upregulated in malignant biopsies	Fold change	*p* value	BH corrected *p* value	Downregulated in malignant biopsies	Fold change	*p* value	BH corrected *p* value
hsa-miR-375	1.80	0.0056	0.5111				
hsa-miR-99a-5p	1.25	0.0302	0.8324				
hsa-miR-19a-3p	1.08	0.0383	0.8324				
hsa-miR-16-2-3p	1.21	0.0482	0.8324				

Supplementary table 5. Dysregulated miRNAs between samples from TRUSbx patients with benign versus malignant outcomes.
